# Early neurological deterioration in patients with minor stroke: A single-center study conducted in Vietnam

**DOI:** 10.1371/journal.pone.0323700

**Published:** 2025-05-19

**Authors:** Dung Tien Nguyen, Ton Duy Mai, Phuong Viet Dao, Hung Tran Ha, Marco Fabus, Melanie Fleming, Minh Cong Tran

**Affiliations:** 1 Bach Mai Stroke Center, Bach Mai Hospital, Hanoi, Vietnam; 2 Vietnam National University-University of Medicine and Pharmacy, Hanoi, Vietnam; 3 Hanoi Medical University, Hanoi, Vietnam; 4 Nuffield Department of Clinical Neuroscience, University of Oxford, Oxford, United Kingdom; UCSF: University of California San Francisco, UNITED STATES OF AMERICA

## Abstract

A minor ischemic stroke is associated with a higher likelihood of poor clinical outcomes at 90 days when there is early neurological deterioration (END). The objective of this case-control study conducted in a comprehensive stroke facility in Vietnam is to examine the frequency, forecast, and outcomes of patients with END in minor strokes. The study employs a descriptive observational design, longitudinally tracking patients with minor strokes admitted to Bach Mai Hospital’s Stroke Center between December 1, 2023, and August 31, 2024. Hospitalized within 24 hours of symptom onset, minor stroke patients with National Institutes of Health Stroke Scale (NIHSS) scores ≤ 5 and items 1a, 1b, and 1c on the NIHSS scale, each equal to 0, were included in the study. The primary measure of interest is the END rate, defined as a rise of 2 or more points in the NIHSS score during the first 72 hours after admission. We conduct a logistic regression analysis to identify forecasting factors for END. Out of 839 patients, 88 (10.5%) had END. In the END group, we found that most patients had complications within the first 24 hours of stroke, accounting for 43.2%; the 24 – 48-hour window accounted for 35.2%, and the 48 – 72-hour window accounted for 21.6%. END was associated with a higher likelihood of poor outcomes (mRS 2 – 6) at discharge (OR = 22.76; 95% CI 11.22 – 46.20; p < 0.01), 30 days post-stroke(OR = 24.38; 95% CI 14.40 – 41.29; p < 0.01), and 90 days post-stroke (OR = 21.74; 95% CI 12.63 – 37.43; p < 0.01). Some of the prognostic factors for END were admission NIHSS score (OR = 1.24; 95% CI 1.03 – 1.49; p = 0.02), admission systolic blood pressure greater than 150mmHg (OR = 1.70; 95% CI 1.03 – 2.81; p = 0.04), admission blood glucose (OR = 1.07; 95% CI 1.01 – 1.14; p = 0.02), reperfusion therapy (OR = 3.35; 95% CI 1.50 – 7.49; p < 0.01), use of antiplatelet monotherapy (OR = 3.69; 95% CI 2.24 – 6.08; p < 0.01), internal capsule infarction (OR = 2.54; 95% CI 1.37 – 4.71; p < 0.01), hemorrhagic transformation (OR = 5.72; 95% CI 1.07 – 30.45; p = 0.04), corresponding extracranial carotid artery occlusion (OR = 4.84; 95% CI 1.26 – 18.65; p = 0.02), and middle cerebral artery occlusion OR = 3.06; 95% CI 1.29 – 7.30; p = 0.01). END in minor stroke patients accounts for 10.5% and is a risk factor for poor neurological outcomes. Admission NIHSS score, higher systolic blood pressure, admission blood glucose, reperfusion therapy, use of antiplatelet monotherapy, internal capsule infarction, hemorrhagic transformation, corresponding extracranial carotid artery occlusion, and middle cerebral artery occlusion were some of the prognostic factors for END in our observational study.

## Introduction

Minor stroke patients constitute between 30% and 57.7% of the total ischemic stroke population [1–8]. The prevailing agreement among clinicians is that a minor stroke is characterised as happening abruptly when the Neurological Impairment Health Status Scale (NIHSS) score does not exceed 5 points, with the awareness items 1a, 1b, and 1c on the NIHSS scale all registering 0 [9–14]. Early neurological deterioration (END) indicates that the patient exhibits worsening focal neurological impairments, accompanied by a rise of 2 or more points in the NIHSS score during the first 72 hours of the stroke [8,15–19]. In previous studies, although patients with minor strokes initially experience END, between 21.4% and 46% exhibit neurological function results, as assessed by the mRS 2 – 6 scale, by day 90 [20–23]. Multiple studies investigating diverse risk factors that forecast adverse outcomes after 90 days have established that END is a critical risk factor [7,15,24–27]. Therefore, evaluating the risk of END in patients with minor strokes at the earliest stages is crucial for developing an effective treatment approach and minimising the probability of negative outcomes by day 90 [8].

In Vietnam, a rise of 4 points or more in the NIHSS during the first 24 hours was identified as a valid forecast of worse clinical outcomes by the 90th day in cases of mild strokes [23]. Nonetheless, this study was deficient in a definitive diagnosis of END, and the definition for a small stroke, based solely on a single instance of NIHSS below 4 points, was insufficient. Therefore, it is essential to pursue more studies on patients with minor strokes. Due to the limited resources for stroke care in Vietnam [6], the objective is to ascertain certain forecast factors that influence END, and hence identify potential targets for intervention.

In this study, we present a cohort of patients who experienced a minor stroke to determine the frequency of END and to examine their clinical, brain imaging, and treatment characteristics to identify predictive factors for END using logistic regression analysis and clinical outcomes on the mRS within a single stroke centre in Vietnam. This is the first reported study about END in minor stroke patients in Vietnam It also contributes to the national guideline for minor stroke treatment in low-middle-income countries with a lack of medical resources.

## Materials and methods

### Study design and patient eligibility

We deliberately structured the study as an observational, descriptive, longitudinal investigation. All patients included in the trial were required to satisfy the following criteria: (1) an ischemic stroke diagnosis, as the AHA recommended [28,29]; (2) an NIHSS score of less than or equal to 5 points, with items 1a, 1b, and 1c on the NIHSS scale all scoring 0. (3) hospitalized within 24 hours following the last known normal. (4) The patient agreed to participate in the research and signed confirmation in informed consent. In case the patient does not communicate with Aphasia, the family (including a first-class blood relationship with patients such as wives, parents or legal representatives) will be replaced. Exclusion criteria for the study included: (1) a pre-stroke mRS score of 2 points or higher; (2) women who were pregnant or breastfeeding; and (3) those with any other brain damage such as traumatic brain injury, intracerebral hemorrhage, subarachnoid hemorrhage, or brain tumor.

The study’s location was the Stroke Center at Bach Mai Hospital in Hanoi, Northern Vietnam. The study ran from December 1, 2023, to August 31, 2024.

All patients with acute stroke within the first 24 hours of enrollment in the study were admitted to our stroke center in the acute stroke unit. Patients were reassessed regularly every 6 hours or whenever abnormalities occurred according to hospital standards for patients with emergency conditions.

The use of antiplatelets at our center follows the recommendations of AHA and ESO for cases of ischemic stroke with NIHSS ≤ 3 points in the first 24 hours, which will be used with dual antiplatelets (aspirin combine clopidogrel) maintained for 21 days (can be extended to a maximum of 90 days if there is symptom intracranial stenosis) and then switched to single antiplatelets according to the results of the 2 studies CHANCE and POINT [10,11,15,30]. According to the THALES study, in cases of patients with ischemic stroke with NIHSS ≤ 5 points, the use of ticagrelor combine aspirin for a maximum of 30 days can be considered, but there will be a higher risk of bleeding. Therefore, in cases of IS with NIHSS 4 – 5 points, clinicians consider using fibrinolytics or dual or single antiplatelets according to the patient’s individualization. Minor stroke patients admitted within the first 4.5 hours with disabling symptoms are indicated for thrombolytic therapy according to the recommendations of AHA and ESO [10,11].

Following Decision No. 4837/BM-HDDD, the Ethics Committee of Bach Mai Hospital approved the study procedure for this paper. Before participation, all patients and their families received a comprehensive explanation of the study and provided their informed consent. The study protocol was published elsewhere in [31].

### Data collection

We included all patients with a minor acute ischemic stroke who satisfied all the inclusion criteria without any specified exclusion criteria. The American Stroke Association’s latest guidelines guided the treatment of every patient with a minor ischemic stroke, which included a comprehensive stroke diagnosis and evaluation based on standard procedures [10,29].

The study data collected from all patients as clinical variables (including: age, sex, systolic blood pressure at admission, diastolic blood pressure at admission, symptoms outside the NIHSS scale (vertigo, horizontal diplopia, vertical diplopia, other diplopia, acute hearing loss), have weak legs or not, have disabling neurological deficit or not, dominant hemisphere lesion, window of admission (<3 hours; 3–4.5 hours; 4.5–6 hours; 6–12 hours; 12–24 hours), risk factors for ischemic stroke (hypertension; diabetes; smoking; previous coronary artery disease/myocardial infarction; history of ischemic stroke/transient ischemic attack; previous mRS; atrial fibrillation/flutter; hypercholesterolemia; obesity; heart failure; mechanical heart valve; bioprosthetic heart valve; TOAST classification) (see S1 Appendix in Supplementary material); paraclinical variables (including: platelets, INR, fibrinogen, aPTTs, cholesterol, HDL-C, LDL-C, triglycerides, urea, creatinine, GOT, GPT, blood sugar at admission, electrocardiogram, throac echocardiogram) and cerebrovascular imaging (including: number of infarct locations, infarct- territory arteris, infarct location, haemorhage transformation, related intracranial artery stenosis/occlusion, related carotid artery stenosis/occlusion); and treatment characteristics variables (thrombolytic therapy, mechanical thrombectomy, antithrombotic drugs (antiplatelet or anticoagulant), statin therapy, diabetes treatment, hypertension treatment), number of days in hospital.

### Follow-up and assessment of endpoints

The END rate, a clinical condition characterized by a gradual decline in neurological function and a two or more point increase in the NIHSS score within 72 hours of symptom onset compared to admission, served as the primary outcome variable. The secondary outcome variables consisted of prognostic factors of END determined by logistic regression analysis and clinical outcomes measured on the mRS scale [32] (see S1 Appendix in Supplementary material).

### Statistical methods

The threshold for a statistically significant difference was a p-value less than 0.05. We used a t-test as the comparison method for normally distributed continuous data. We employed the Kruskal-Wallis test as the comparison method for continuous variables that did not follow a normal distribution. We analyzed categorical variables using the chi-square test. We applied the multivariate logistic regression model after identifying variables associated with demographic, clinical, paraclinical, cerebrovascular imaging, and treatment characteristics that differed between the two groups, END and No-END. Data were analysed using SPSS v16.o (IBM Inc, USA).

After collecting comprehensive data on the patients, the study categorized them into two groups: one with END and another with No-END. We compared the two groups’ demographic, clinical, paraclinical, cerebrovascular image, and treatment characteristics to forecast and find the unique features. Therefore, a need for a p-value less than 0.2 is important. This helped us choose the best variables for the prognostic model. To identify prognostic determinants of END, we performed a logistic regression analysis based on these various features, with END serving as the outcome variable. Building upon that, we developed the END prognostic model.

## Results

**Fig 1:**
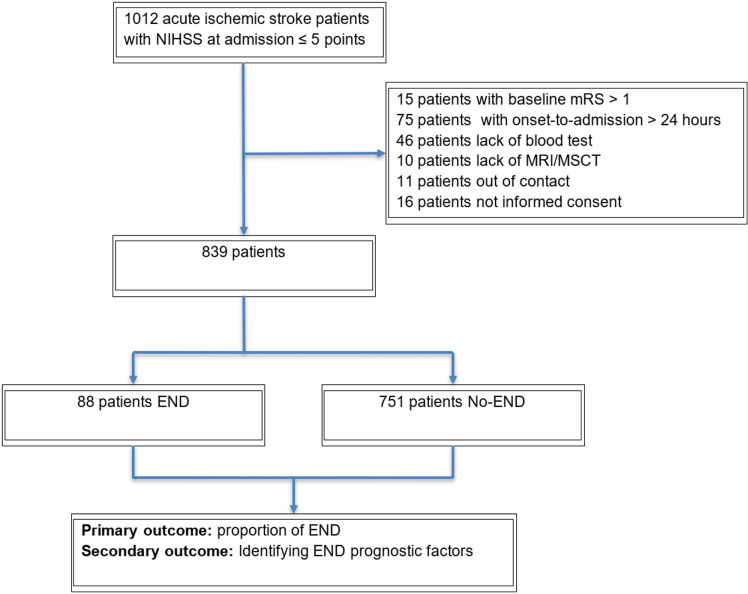
Flow chart of the study from top to bottom. Starting with the number of patients included in the study, with the exclusion criteria, and the number of patients in both END and No-END groups.

Our study included 839 individuals who satisfied the inclusion and exclusion criteria (Fig 1). Out of 839 patients, 88 (10.5%) had END. In the END group, we found that most patients had complications within the first 24 hours of the disease, accounting for 43.2% The 24 – 48-hour window accounted for 35.2%, and the 48 – 72-hour window accounted for 21.6% of patients (S4 Table in Supplementary data). In the END patient cohort, 42.1% exhibited an increase of 2 points in NIHSS score at the time of END; 38.6% had an increase of 4 points or more, while the group with a 3-point rise had the lowest proportion at 19.3% (S5 Table in Supplementary data).

Patients with END had more extended hospital stays and higher mRS scores at discharge and on the 30th and 90th days of illness (Table 1). END was associated with a higher likelihood of poor neurological outcomes (mRS 2–6) at discharge (OR = 22.76; 95% CI 11.22 – 46.20; p < 0.01), 30th day of disease (OR = 24.38; 95% CI 14.40 – 41.29; p < 0.01), and 90th day of disease (OR = 21.74; 95% CI 12.63 – 37.43; p < 0.01) (S3 Fig in Supplementary data).

**Table 1:** Clinical, paraclinical, and therapeutic characteristics in the END and No END cohorts.

	Total N = 839 100%	END N = 88 10.5%	No END N = 751 89.5%	p	Note
**Patient characteristics**					
Age (IQR)	65 (57-72)	65 54-71	65 57-72	0.81	θ, ¥
Male, n (%)	**544** **64.8**	**51** **(58.0)**	**493 (65.6)**	**0.15**	£,¤
Initial SBP, mmHg, mean ± sd	**152.7 ± 23.4**	**156.88 ± 22.74**	**152.19 ± 23.45**	**0.07**	θ, ¥ ¤
Initial DBP, mmHg, mean ± sd	**88.3 ± 14.01**	**90.16 ± 14.79**	**88.05 ± 13.91**	**0.18**	θ, ¥ ¤
Initial SBP ≥ 150mmHg n %	**453** **54.0**	**59** **67.0**	**394** **52.5**	***<0.01***	£ ¤
NIHSS, IQR	**3 (2-4)**	**3 (2-4)**	**3 (2-3)**	***<0.01***	θ, ¥ ¤
***Risk of stroke***					
Hypertension n (%)	582 69.4	69 78.4	513 68.3	**0.05**	£ ¤
Atrial Fibrillation/Atrial Flutter n (%)	24 2.9	1 1.1	23 3.1	0.501	£
Diabetes n (%)	195 23.2	21 23.9	174 23.2	0.88	£
High blood cholesterol n (%)	59 7.0	8 9.1	51 6.8	0.43	£
Smoking n (%)	198 23.8	23 26.1	176 23.4	0.57	£
Overweight/Obese (BMI >=25) n %	154 18.4	18 20.5	136 18.1	0.59	£
***Blood test***					
Platelets mean ± sd	**264.2 ± 69.1**	**276.15 ± 76.24**	**262.81 ± 68.16**	**0.12**	θ, ¥ ¤
Fibrinogen mean ± sd	**3.41 ± 0.76**	**3.55 ± 0.82**	**3.40 ± 0.76**	**0.08**	θ, ¥ ¤
Cholesterol total mean ± sd	**4.90 ± 1.18**	**5.10 ± 1.32**	**4.88 ± 1.16**	**0.09**	θ, ¥ ¤
LDL-C mean ± sd	**2.75 ± 0.95**	**2.91 ± 1.07**	**2.73 ± 0.93**	**0.11**	θ, ¥ ¤
Glucose on admission mean ± sd	**7.8 ± 3.3**	**8.71 ± 4.62**	**7.68 ± 3.14**	**0.04**	θ, ¥ ¤
Atrial Fibrillation/Atrial Flutter on admission n (%)	23 (2.7)	2 (2.3)	26 (3.5)	0.76	£
***Echocardiography***					
Moderate or severe mitral stenosis n (%)	6 0.7	0 0	6 0.8	1.0	£
Heart failure n (%)	28 3.3	5 5.7	23 3.1	0.20	£
***TOAST classification***					
Large artery atherosclerosis n (%)	114 13.6	15 17.0	99 13.2	0.32	£
Cardioembolism n (%)	25 3.0	1 1.1	24 3.2	0.50	£
Small artery occlusion n (%)	476 56.7	47 53.4	429 57.1	0.51	£
Stroke of other determined cause n (%)	7 0.8	1 1.1	6 0.8	0.54	£
Stroke of undetermined cause n (%)	217 25.9	24 27.3	193 25.7	0.75	£
***Treatment***					
Reperfusion treatment n (%)	**36** **4.3**	**15** **17.0**	**21** **2.8**	***<0.01***	£ ¤
rtPA n (%)	35 4.2	13 14.8	22 2.9	NA	£
Mechanical thrombectomy n (%)	2 0.2	2 2.3	0 0	NA	£
Antiplatelet monotherapy n (%)	**223** **26.6**	**51** **58.0**	**172** **22.9**	***<0.01***	£ ¤
Antiplatelet dual therapy n (%)	**585** **69.7**	**36** **40.9**	**549** **73.1**	***<0.01***	£ ¤
Anticoagulation n (%)	29 3.5	1 1.1	28 3.7	0.35	£
Number of days in hospital Day, IQR	**3** **2-4**	**3** **3-4**	**3** **2-4**	**0.01**	φ, € ¤
mRS score at discharge IQR	**1** **1-2**	**3** **2-3**	**1** **1-2**	***<0.01***	φ, € ¤
mRS score at 30 days IQR	**0** **0-1**	**2** **1-3**	**0** **0-1**	***<0.01***	φ, € ¤
mRS score at 90 days IQR	**0** **0-1**	**2** **1-3**	**0** **0-1**	***<0.01***	φ, € ¤

θ - normally distributed continuous data, φ - nonnormally distributed continuous data.

¥ - t-test; € - Kruskal-Wallis test; £ - chi-square test.

¤ - variable that satisfies p < 0.2.

PFO – Patent foramen ovale; rtPA – recombinant tissue plasminogen activators; mRS - Modified Rankin Scale; INR - International Normalized Ratio; TIA - transient ischaemic attack; SBP – systolic blood pressure; DBP – diastolic blood pressure; NIHSS – national institute of health stroke scale; BMI – body mass index; aPTT – activated partial thromboplastin time; HDLC – high-density lipoprotein; LDLC – low-density lipoprotein; GOT – glutamic oxaloacetic transaminase; GPT – glutamic pyruvic transaminase; TOAST – trial of ORG 10172 in acute stroke treatment.

### Clinical features, blood tests, brain images, and treatment characteristics

Compared to the no-END group, the END group exhibited a greater proportion of patients with admission systolic blood pressure above 150mmHg (67.0% and 52.5%, p < 0.01, respectively), increased admission NIHSS scores (IQR 3 (2 – 4) and 3 (2 – 3), p < 0.01, respectively), heightened admission blood glucose levels (8.71 ± 4.62 and 7.68 ± 3.14, p = 0.04, respectively), a larger proportion of patients experience reperfusion therapy (including thrombolysis and mechanical thrombectomy) (17.0% and 2.8%, p < 0.01, respectively), a higher proportion of patients on single antiplatelet therapy (58% and 22.8%, p < 0.01, respectively), and a lower proportion of patients on dual antiplatelet therapy (40.9% and 73.1%, p < 0.01, respectively) compared to the no-END group (S1 Table and Supplementary material).

In the study population of 839 patients, the rate of CTA to evaluate intracranial arteries was 27.9%, whereas MRA was 72.1% (S1 Appendix in Supplementary material). Of them, 725/839 (86.4%) patients had ischemic stroke lesions visible on the film (therefore defining the corresponding cerebral vasculature), with 73 patients in the END group and 652 patients in the No-END group (Table 2). We found that the END group had a higher rate of ischemic stroke site in the internal capsule, hemorrhagic transformation rate, extracranial carotid artery occlusion rate, middle cerebral artery occlusion rate, and intracranial artery occlusion than (Table 2).

**Table 2:** Characteristics of cerebral vascular imaging.

	Total N = 725 100%	END N = 73 10.5%	No END N = 652 89.5%	p	note
*Cerebral infarction sites*					
Thalamus n (%)	90 (12.4)	8 (11.0)	82 (12.6)	0.69	£
Internal capsule n (%)	**91 (12.6)**	**17 (23.3)**	**74 (11.3)**	**< 0.01**	£ ¤
Caudate nucleus n (%)	**34 (4.7)**	**7 (9.6)**	**27 (4.1)**	**0.07**	£ ¤
Lentiform nucleus n (%)	182 (25.1)	19 (26.0)	163 (25.0)	0.85	£
Insular n (%)	39 (5.1)	6 (7.7)	33 (4.8)	0.28	£
Corona radiata n (%)	**220 (30.3)**	**28 (38.4)**	**192 (29.4)**	**0.12**	£ ¤
Corpus callosum n (%)	1 (0.1)	1 (1.4)	0	0.10	£
Temporal lobe n (%)	72 (9.9)	10 (13.7)	62 (9.5)	0.26	£
Frontal lobe n (%)	53 (7.3)	5 (6.8)	48 (7.4)	0.87	£
Parietal lobe n (%)	35 (4.8)	3 (4.1)	32 (4.9)	1.0	£
Occipital lobe n (%)	57 (7.9)	4 (5.5)	53 (8.1)	0.43	£
Brainstem n (%)	121 (15.8)	10 (12.8)	111 (16.2)	0.52	£
Cerebellum n (%)	48 (6.3)	1 (1.3)	47 (6.8)	0.08	£
Hemorrhagic transformation n (%)	**6 (0.8)**	**2 (2.7)**	**4 (0.6)**	**0.03**	£ ¤
*Corresponding extracranial ICA Characteristics*					
Stenosis 50–99% n (%)	32 (4.4)	4 (5.5)	28 (4.3)	0.55	£
Occlusion n (%)	**11 (1.5)**	**4 (5.5)**	**7 (1.1)**	**0.02**	£ ¤
*Corresponding Intracranial Artery Characteristics*					
Stenosis 50–99% n (%)	57 (7.9)	4 (5.5)	53 (8.1)	0.43	£
Occlusion n (%)	**60 (8.3)**	**10 (13.7)**	**50 (7.7)**	**0.06**	£ ¤
*Corresponding intracranial artery occlusion site*					
ICA n (%)	14 (1.9)	0	14 (2.1)	0.38	£
MCA n (%)	**33 (4.6)**	**9 (12.3)**	**24 (3.7)**	**< 0.01**	£ ¤
BA n (%)	6 (0.8)	1 (1.4)	5 (0.8)	0.47	£

£ - Chi-square test; ¤ - a variable that satisfies p < 0.2 and has a clinical value that may be related to END.

ICA: Internal Carotid Artery; ACA: Anterior Cerebral Artery; MCA: Middle Cerebral Artery; PCA; Posterior Cerebral Artery; BA: Basilar Artery.

Among our 6 cases of hemorrhagic transformation, two patients were classified as END and four patients as non-END. Among the 2 END cases, one was classified as parenchymal hematoma 2 according to the ECASS trial after using alteplase, and one progressed to spontaneous hemorrhagic infarction 1, with both instances classified as symptomatic intracerebral hemorrhage. None of the four no-END patients had reperfusion treatment; three were classified to hemorrhagic infarction 1 and one to hemorrhagic infarction 2.

**Fig 2:**
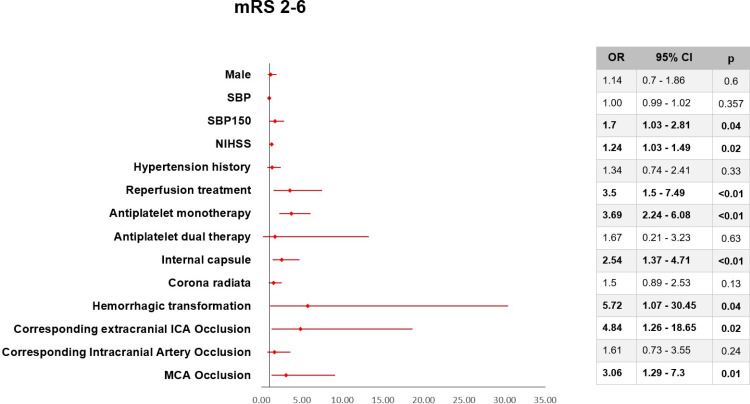
Multivariate logistic regression analysis of some prognostic factors for END. SBP - systolic blood pressure on admission; SBP150 – systolic blood pressure on admission above 150mmHg; NIHSS - national institute of health stroke scale; ICA - internal carotid artery; MCA - middle cerebral artery.

We conducted a multivariate logistic regression analysis to identify potential prognostic factors for END in minor stroke, confirming NIHSS score at admission, SBP more than 150mmHg at admission, blood glucose at admission, reperfusion therapy (including thrombolysis and mechanical thrombectomy), use of single antiplatelet drugs, ischemic stroke at the internal capsule location, hemorrhagic transformation (Fig 2).

## Discussion

Despite the early symptoms of a minor stroke being mild, the presence of an END elevates the likelihood of an unfavourable outcome [23,33,34]. END events commonly occur within the first hours, a day, or two after symptom onset [34]. The cause of END is unknown. However, it might be altered cerebral perfusion, endotoxin excitotoxicity, inflammatory agents, edema resulting in neurotransmission loss, or progressive thrombosis [35]. Still, END in individuals with acute ischemic stroke who have initially minor clinical symptoms is a difficult situation that need cautious observation and timely treatment. However, the criteria from previous studies for END are inconsistent (the NIHSS score might increase merely because the patient has weaker motor function, but it can also decrease) and vary in progression across time (24 hours to 3 weeks or throughout hospitalization) [7,8,15,17,26,27,36]. The variability of these categories makes comparing research somewhat challenging.

The definition of END includes two criteria: (1) the timing of END’s appearance and (2) an increase in NIHSS scores. Regarding time, some authors set a threshold for the first 24 hours, 72 hours, seven days, or even three weeks, as in Ju Yi’s study [7,8,12,13,15–19,24,26,37,38]. In some other studies, they employ the time requirement of the first 24 hours in investigations of the minor stroke while the onset is connected with a bigger vascular occlusion, regardless of whether the patient has or does not have a larger artery obstruction [27,38,39]. With the extra NIHSS score criteria, another study discovered that cutoff 2 was the most commonly utilized threshold and best-assured consistency across trials [36]. This is the basis for the END definition’s criterion of two or more points.

In our study, the END rate was 88 out of 839 (10.5%), lower than that Xiaohua Xie reported at 16.4% and Avan Sabir Rashid at 20.8% [18,37]. This might be because Xiaohua Xie’s study defined the END criteria as a rise of two or more points in the NIHSS score within seven days. Furthermore, Avan Sabir Rashid’s study only enrolled 101 individuals, but the population in our study included 839 patients who were larger than others. Other investigations reported an END rate ranging from 2.8 to 48.7% [8]. This considerable range might be attributed to discrepancies in the classification of minor stroke, with some research employing the NIHSS criterion of 3 or 5 points. Recent studies indicate that an NIHSS threshold of 5 points is deemed more suitable, as both groups exhibit comparable risks of overall vascular events (including mortality, recurrent stroke, and myocardial infarction) and complication rates (gastrointestinal bleeding and deep vein thrombosis of the lower extremities) [33,40–42]. The AHA and ESO guidelines recommend using an NIHSS criterion of ≤ 5 points [10,11]. Our study used the NIHSS threshold of ≤ 5 points as the minor stroke. Our study differs from previous research on END in minor stroke with NIHSS ≤ 3 by Ju Yi (2013), Xingyang Yi (2016), Dezhi Liu (2016), Sam Min Sung (2020), and Zuowei Duan (2020) [8,16,19,26,43].

Our study identified several prognostic factors for END, including NIHSS score at admission, systolic blood pressure at admission above 150mmHg, blood glucose at admission, reperfusion therapy, use of antiplatelet monotherapy, internal capsule ischemic stroke, hemorrhagic transformation, corresponding extracranial carotid artery occlusion, and corresponding middle cerebral artery occlusion. NIHSS score at admission was a prognostic factor for END (OR = 1.24; 95% CI 1.03 – 1.49; p = 0.02), comparable to the study of authors Sam Min Sung (OR = 1.4; 95% CI 1.13 – 1.73; p < 0.01), Xiaohua Xie (OR = 1.29; 95% CI 1.20 – 1.28; p < 0.01), Hongli Zhang (OR = 1.99; 95% CI 1.049 – 3.772; p = 0.04) [8,12,37]. A high NIHSS score corresponds to a larger region of cerebral infarction. As a result, the chance of cerebral infarction advancement increases [12].

In our study, high systolic blood pressure at admission was associated with END (OR = 1.70; 95% CI 1.03 – 2.81; p = 0.04), comparable to author Zuowei Duan (OR = 2.71; 95% CI 1.012 – 7.253; p < 0.01) [15]. According to the pathophysiological process, cerebral perfusion is significantly regulated by the brain’s autoregulatory system based on blood pressure, particularly systolic blood pressure. In the setting of stroke, the higher the blood pressure, the greater the danger of disrupting the balance of the autoregulatory system, compromising cerebral perfusion, putting the penumbra at risk of evolving into an infarction region, and worsening clinical signs [44].

Our findings indicated that a history of diabetes was not linked with END, although blood glucose levels were (OR = 1.07; 95% CI 1.01 – 1.14; p = 0.02). This may be explained by the fact that patients with a history of diabetes but well-controlled, not high blood glucose, have not associated with END. This finding was also reported in investigations by authors Xingyang Yi and Avan Sabir Rashid [16,18]. Hyperglycemia raises lactate levels in the brain, which may increase the risk of necrosis in regions with poor cerebral perfusion [45]. Hyperglycemia raises the risk of endothelial damage to cerebral blood vessels, particularly in brain parts injured by infarction, resulting in more widespread infarctions.

Among the six patients of hemorrhage transformation, only 1 case occurred post-thrombolytic therapy and resulted in sICH; the other five instances transformed due to the natural development of the illness. Ischemic stroke with hemorrhagic transformation, according to author Sam Min Sung, was a factor increasing the risk of END with OR = 3.80 (95% CI 1.38 – 10.45; p = 0.01) [8]. One reason may be due to the integrity of the blood-brain barrier and the structure of the neurovascular unit being broken. Occlusion of the corresponding extracranial ICA segment and MCA occlusion as described by Sam Min Sung (OR = 2.56; 95% CI 1.36 – 4.82; p < 0.01 for intracranial occlusion), N.Boulenoir, Pierre Seners (OR = 16.0; 95% CI 5.7 – 44.9; p < 0.01 and OR = 4.2; 95% CI 1.8 – 9.4; p < 0.01 respectively) [8,27,38]. The elements listed above, intracerebral hemorrhage and cerebral artery occlusion, have a direct impact on cerebral perfusion and brain cell death. Consequently, they worsen the ischemic stroke. Our study discovered infarction in the internal capsule site as an independent prognostic factor for END, presumably because the internal capsule area contains many motor nerve fibers and a high concentration [46]. Our analysis found that internal capsule ischemic stroke increased the incidence of END with an OR = 2.54 (95% CI 1.37 – 4.71; p < 0.01). Our findings align with those of Takase and Tomoyuki Ohara: lacunar ischemia at the perfusion location of the lenticulostriate artery as a prognostic indicator for progressive motor paralysis [47,48].

According to the AHA and ESO recommendations, dual antiplatelet therapy (aspirin combined with clopidogrel) is recommended for ischemic stroke with an NIHSS score of 3 or less [10,11]. However, if the NIHSS is 4–5, the recommendation is of a lower level and comes from the THALES study (assessing the role of DAPT with aspirin combined with ticagrelor), and the risk of bleeding will be higher [49]. In our study,patients who used antiplatelet monotherapy (OR = 3.69; 95% CI 2.24 – 6.08; p < 0.01) and reperfusion treatment (OR = 3.5; 95% CI 1.50 – 7.49; p < 0.01) had a greater risk of END compared to other groups, as reported by Fan He and Yu Cui [7,15]. According to Fan He’s research, the minor stroke group that used dual antiplatelet medications (aspirin and clopidogrel) had an END rate of only 2.8%, compared to 5.8% in the group that used single antiplatelet drugs [15]. The AHA and ESO guidelines recommend from CHANCE and POINT trials for the use of dual antiplatelet agents with clopidogrel in RCT studies for the minor stroke group with NIHSS ≤ 3 or high-risk TIA, and THALES for the use of dual antiplatelet agents with ticagrelor in the minor stroke group with NIHSS ≤ 5 points or high-risk TIA with larger artery stenosis, but with a higher risk of bleeding [29,30,50]. Recent trials suggest that dual antiplatelet therapy is more effective than single antiplatelet therapy in reducing the risk of END (risk difference −1.9%; 95% CI −3.6 to −0.2; P = 0.03) in minor to moderate ischemic stroke, similar to ATAMIS, without increasing the risk of bleeding [51]. A secondary analysis from ARAMIS found that antiplatelet dual therapy treatment was more effective than rtPA in lowering the incidence of END in minor stroke patients with high SBP [7]. Among these 35 instances, only one exhibited hemorrhagic transformation, further substantiating the safety of reperfusion treatment in patients with minor strokes. In our study population, reperfusion therapy was ineffective, as the incidence of END patients in the reperfusion therapy group exceeded that in the no-END group, with an OR = 3.50 (95% CI 1.5 – 7.49; p < 0.01). The limited size of the reperfusion treatment group in our research cohort may account for this: thrombolysis was provided to 35 of 839 patients (4.2%), and mechanical thrombectomy was conducted on 2 of 839 patients (0.2%). This metric also represents the real clinical context of reperfusion therapy in individuals with minor strokes in Vietnam. The current guidelines for this patient cohort are ambiguous. Particularly for minor stroke with large artery occlusion, mechanical thrombectomy requires further data from randomized controlled trials (RCTs). Two significant RCTs are currently in progress: ENDOLOW (NCT04167527) and MOSTE (NCT03796468) [52,53].

Limitations of the study include (1) This is an observational study, which raises the possibility of sample selection bias. (2) A single-centre research does not guarantee representativeness and universality, which may lack generalizability beyond the Vietnamese population. (3) The study did not evaluate some other factors related to END like previous studies, such as intracranial stenosis due to low frequency in the study population, subdividing into mild, moderate, and severe stenosis, some advanced imaging characteristics (cerebral perfusion parameters: rCBV, infarct core volume, penumbra volume, etc.), some biological markers such as 20-HETE, pNFL, and no new findings at the molecular or metabolic level *[*12,16,17,39]. (4) We observed the impact of DAPT, SAPT, or alteplase on the treatment outcomes of END and mRS at day 90 without assessing adherence and dosing. Therefore, our study results would be less convincing. (5) High admission blood pressure here refers to cases where systolic blood pressure upon admission has a value greater than 150mmHg, we observed that it is associtated to END. However, our study has a limitation that it does not evaluate the blood pressure management of all patients, it may have impacted the outcomes. (6) Our study only assessed the short-term effects of END and treatment outcomes after a short period of 90 days, without addressing their long-term effects such as cognitive impairment and quality of life.

## Conclusion

END in minor stroke patients is a risk factor for poor neurological outcomes. Admission NIHSS score, admission systolic blood pressure greater than 150mmHg, admission blood glucose, reperfusion therapy, use of single antiplatelet therapy, internal capsule infarction, hemorrhagic transformation, corresponding extracranial carotid artery occlusion, and middle cerebral artery occlusion were some of the prognostic factors for END in our observational study.

## Supporting information

S1 Appendix Supplementary material. (DOCX)

S1 File Data MinorStroke. (XLSX)

S2 File Inclusivity in Global Research Questionnaire. (DOCX)
