# Supplementary material for: Early neurological deterioration in patients with minor stroke: A single-center study conducted in Vietnam
Source: PLoS One. 2025 May 19;20(5):e0323700. doi: 10.1371/journal.pone.0323700 (PMC12088008; doi:10.1371/journal.pone.0323700)
Supplement: S1 Appendix — (DOCX) [file pone.0323700.s001.docx]

**S1 Appendix SUPPLEMENTARY MATERIAL**

**General characteristics of the study population**

Our study included 839 individuals who satisfied the inclusion and exclusion criteria (see **Figure 1**). Out of 839 patients, 88 (10.5%) had END. Patients with END had more extended hospital stays and higher mRS scores at discharge and on the 30th and 90th days of illness (**Table 1**).


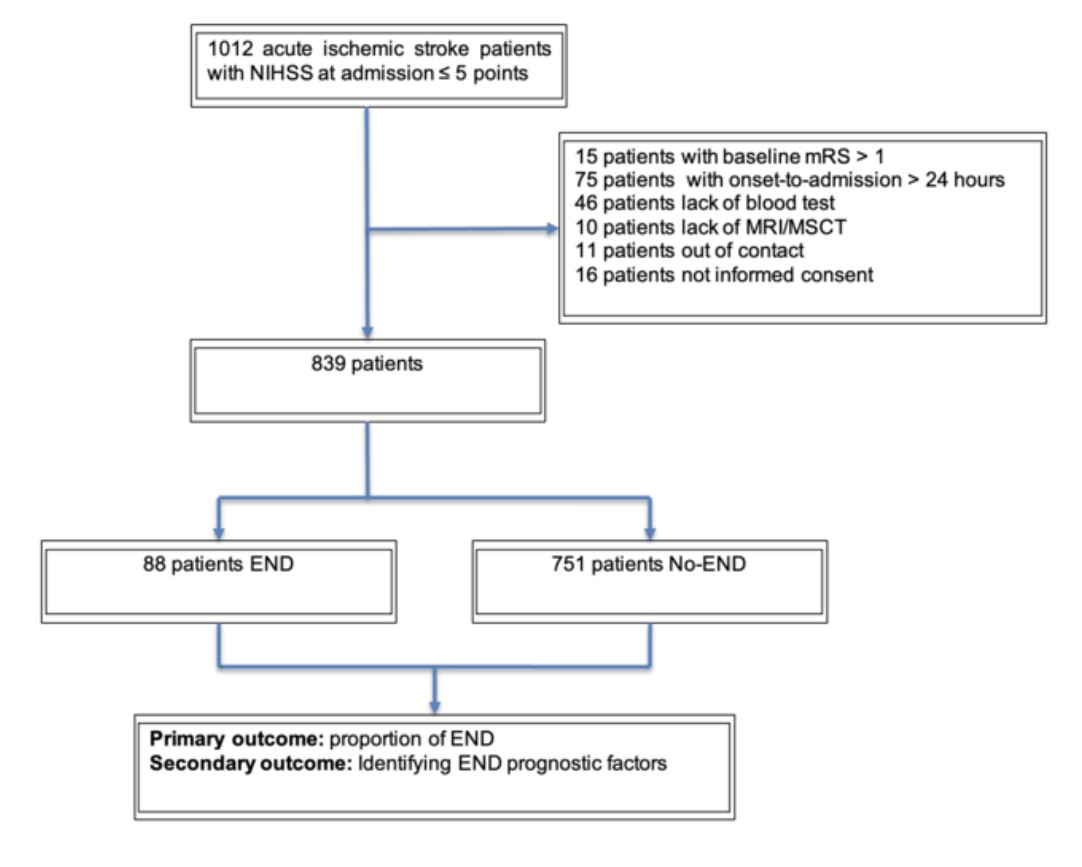


**Fig 1. Flow chart of the study from top to bottom**. Starting with the number of patients included in the study, with the exclusion criteria, and the number of patients in both END and No-END groups

**Clinical features, blood tests, and treatment characteristics**

Through univariate analysis of clinical characteristics, blood tests, echocardiography, and treatment variables, we discovered that the proportion of patients with systolic blood pressure at admission greater than 150mmHg, NIHSS score at admission, blood glucose index at admission, reperfusion treatment rate (including thrombolysis and mechanical thrombectomy), and antiplatelet monotherapy use rate were higher than in the No-END group. However, in the END group, the proportion of patients adopting dual antiplatelet treatment was statistically substantially lower than the No-END group (**Table 1**).

**Table 1:** **Clinical, paraclinical, and therapeutic characteristics in the END and No END cohorts**

|  | **Total**  **N=839**  **100%** | **END**  **N=88**  **10.5%** | **No END**  **N =751**  **89.5%** | **p** | **Note** |
| --- | --- | --- | --- | --- | --- |
| **Patient Characteristics** | | | | |  |
| Age  (IQR) | 65 (57-72) | 65  54-71 | 65  57-72 | 0.81 | α, ¥ |
| Male,  n ( %) | 544 (64.8) | 51 (58.0) | 493 (65.6) | **0.15** | £,¤ |
| Initial SBP, mmHg,  mean ± sd | 152.7 ± 23.4 | 156.88 ± 22.74 | 152.19 ± 23.45 | **0.07** | α, ¥ ¤ |
| Initial DBP, mmHg,  mean ± sd | 88.3 ± 14.01 | 90.16 ± 14.79 | 88.05 ± 13.91 | **0.18** | α, ¥ ¤ |
| Initial SBP ≥ 150mmHg  n % | 453  54.0 | 59  67.0 | 394  52.5 | ***<0.01*** | £ ¤ |
| NIHSS,  IQR | 3 (2-4) | 3 (2-4) | 3 (2-3) | ***<0.01*** | α, ¥ ¤ |
| Disabling  n ( %) | 349  41.6 | 41  46.6 | 308  41.0 | 0.32 | £ |
| Dominant hemisphere lesion  n ( %) | 255  30.4 | 27  30.7 | 228  30.4 | 0.95 | £ |
| The time window of hospitalization from the onset | | | | | |
| Initial 3 hours  n % | 100  11.9 | 14  15.9 | 86  11.5 | 0.019 | £ |
| 3-4.5 hours  n % | 88  10.5 | 7  8.0 | 81  10.8 |  |  |
| 4.5-6 hours  n % | 102  12.2 | 19  21.6 | 83  11.1 |  |  |
| 6-12 hours  n % | 202  24.1 | 21  23.9 | 181  24.1 |  |  |
| 12-24 hours  n % | 347  41.4 | 27  30.7 | 320  42.6 |  |  |
| ***Risk of stroke*** | | | | |  |
| Hypertension  n ( %) | 582  69.4 | 69  78.4 | 513  68.3 | **0.05** | £ ¤ |
| Atrial Fibrillation/Atrial Flutter  n ( %) | 24  2.9 | 1  1.1 | 23  3.1 | 0.501 | £ |
| Diabetes  n ( %) | 195  23.2 | 21  23.9 | 174  23.2 | 0.88 | £ |
| High blood cholesterol  n ( %) | 59  7.0 | 8  9.1 | 51  6.8 | 0.43 | £ |
| Smoking  n ( %) | 198  23.8 | 23  26.1 | 176  23.4 | 0.57 | £ |
| Overweight/Obese BMI >=25  n % | 154  18.4 | 18  20.5 | 136  18.1 | 0.59 | £ |
| Coronary artery disease/MI history  n ( %) | 17  2.0 | 2  2.3 | 15  2.0 | 0.70 | £ |
| Heart failure  n ( %) | 19  2.3 | 4  4.5 | 15  2.0 | 0.13 | £ |
| Previous Ischemic stroke/TIA  n ( %) | 100  11.9 | 5  5.7 | 95  12.6 | 0.056 | £ |
| mRS 1  n ( %) | 30  3.6 | 2  2.3 | 28  3.7 | 0.76 | £ |
| ***Blood test*** | | | | |  |
| Platelets  mean ± sd | 264.2 ± 69.1 | 276.15 ± 76.24 | 262.81 ± 68.16 | **0.12** | α, ¥ ¤ |
| INR  mean ± sd | 0.98 ± 0.28 | 0.95 ± 0.084 | 0.98 ± 0.30 | 0.23 | α, ¥ |
| Fibrinogen  mean ± sd | 3.41 ± 0.76 | 3.55 ± 0.82 | 3.40 ± 0.76 | **0.08** | α, ¥ ¤ |
| aPTTs  mean ± sd | 28.92 ± 3.53 | 28.51 ± 3.29 | 28.99 ± 3.56 | 0.25 | α. ¥ |
| Cholesterol total  mean ± sd | 4.90 ± 1.18 | 5.10 ± 1.32 | 4.88 ± 1.16 | **0.09** | α, ¥ ¤ |
| HDL-C  mean ± sd | 1.13 ± 0.39 | 1.18 ± 0.36 | 1.13 ± 0.39 | 0.21 | α, ¥ |
| LDL-C  mean ± sd | 2.75 ± 0.95 | 2.91 ± 1.07 | 2.73 ± 0.93 | **0.11** | α, ¥ ¤ |
| Triglycerid  mean ± sd | 2.44 ± 1.73 | 2.49 ± 1.80 | 2.44 ± 1.72 | 0.77 | α, ¥ |
| Ure  mean ± sd | 5.60 ± 2.67 | 5.65 ± 3.71 | 5.59 ± 2.52 | 0.85 | α, ¥ |
| Creatinin  mean ± sd | 76.47 ± 23.44 | 74.10 ± 25.66 | 76.75 ± 23.16 | 0.32 | α, ¥ |
| GOT  mean ± sd | 26.03 ± 14.85 | 24.15 ± 13.57 | 25.26 ± 14.98 | 0.21 | α, ¥ |
| GPT  mean ± sd | 25.31 ± 21.46 | 22.34 ± 14.27 | 25.66 ± 22.13 | **0.17** | α, ¥ ¤ |
| Glucose on admission  mean ± sd | 7.8 ± 3.3 | 8.71 ± 4.62 | 7.68 ± 3.14 | **0.04** | α, ¥ ¤ |
| Atrial Fibrillation/Atrial Flutter on admission  n ( %) | 23  2.7 | 2  2.3 | 26  3.5 | 0.76 | £ |
| ***Transthoracic echocardiography*** | | | | |  |
| Moderate or severe mitral stenosis  n ( %) | 6  0.7 | 0  0 | 6  0.8 | 1.0 | £ |
| Heart failure  n ( %) | 28  3.3 | 5  5.7 | 23  3.1 | 0.20 | £ |
| PFO  n (%) | 1  0.1 | 0  0 | 1  0.1 | 1.0 | £ |
| ***TOAST Classification*** | | | | |  |
| Large artery atherosclerosis  n ( %) | 114  13.6 | 15  17.0 | 99  13.2 | 0.32 | £ |
| Cardioembolism  n ( %) | 25  3.0 | 1  1.1 | 24  3.2 | 0.50 | £ |
| Small artery occlusion  n ( %) | 476  56.7 | 47  53.4 | 429  57.1 | 0.51 | £ |
| Stroke of other determined cause n ( %) | 7  0.8 | 1  1.1 | 6  0.8 | 0.54 | £ |
| Stroke of undetermined cause  n ( %) | 217  25.9 | 24  27.3 | 193  25.7 | 0.75 | £ |
| ***Treatment*** | | | | | |
| Reperfusion treatment  n ( %) | 36  4.3 | 15  17.0 | 21  2.8 | ***<0.01*** | £ ¤ |
| rtPA  n ( %) | 35  4.2 | 13  14.8 | 22  2.9 | NA | £ |
| Mechanical thrombectomy  n ( %) | 2  0.2 | 2  2.3 | 0  0 | NA | £ |
| Antiplatelet monotherapy  n ( %) | 223  26.6 | 51  58.0 | 172  22.9 | ***<0.01*** | £ ¤ |
| Antiplatelet dual therapy  n ( %) | 585  69.7 | 36  40.9 | 549  73.1 | ***<0.01*** | £ ¤ |
| Anticoagulation  n ( %) | 29  3.5 | 1  1.1 | 28  3.7 | 0.35 | £ |
| Statin n ( %) | 839  100 | 88  100 | 751  100 | 1.0 | £ |
| Number of days in hospital  Day. IQR | 3  2-4 | 3  3-4 | 3  2-4 | **0.01** | β € ¤ |
| mRS score at discharge  IQR | 1  1-2 | 3  2-3 | 1  1-2 | ***<0.01*** | β € ¤ |
| mRS score at 30 days  IQR | 0  0-1 | 2  1-3 | 0  0-1 | ***<0.01*** | β € ¤ |
| mRS score at 90 days  IQR | 0  0-1 | 2  1-3 | 0  0-1 | ***<0.01*** | β € ¤ |

α - normally distributed continuous data, β - nonnormally distributed continuous data

¥ - t-test ; € - Kruskal-Wallis test ; £ - chi-square test

¤ - variable that satisfies p < 0.2.

PFO – Patent foramen ovale; rtPA – recombinant tissue plasminogen activators; mRS - Modified Rankin Scale; INR - International Normalized Ratio; TIA - transient ischaemic attack; SBP – systolic blood pressure; DBP – diastolic blood pressure; NIHSS – national institute of health stroke scale; BMI – body mass index; aPTT – activated partial thromboplastin time; HDLC – high-density lipoprotein; LDLC – low-density lipoprotein; GOT – glutamic oxaloacetic transaminase; GPT – glutamic pyruvic transaminase; TOAST – trial of ORG 10172 in acute stroke treatment.

**Table 2** **Cerebral angiography method**

| ***Cerebral angiography*** | **Total**  **N (%)** | **END**  **N (%)** | **No-END**  **N (%)** | **p** |
| --- | --- | --- | --- | --- |
| MRA  n ( %) | 605  72.1 | 60  68.2 | 545  72.6 | 0.39 |
| CTA  n ( %) | 234  27.9 | 28  31.8 | 206  27.4 | 0.39 |

**Table 3:** **Characteristics of cerebral vascular imaging**

|  | Total  N=725  100% | END  N=73  10.5% | No END  N =652  89.5% | p | note |
| --- | --- | --- | --- | --- | --- |
| *The number of cerebral infarction sites* | | | | | |
| 1  n ( %) | 513  70.8 | 47  64.4 | 466  71.5 | 0.21 | £ |
| 2  n ( %) | 105  14.5 | 12  16.4 | 93  14.3 | 0.62 | £ |
| > 2  n ( %) | 107  14.8 | 14  19.2 | 94  14.3 | 0.26 | £ |
| *Cerebral infarction sites* | | | | | |
| Thalamus  n ( %) | 90  12.4 | 8  11.0 | 82  12.6 | 0.69 | £ |
| Internal capsule  n ( %) | 91  12.6 | 17  23.3 | 74  11.3 | **< 0.01** | £ ¤ |
| Caudate nucleus  n ( %) | 34  4.7 | 7  9.6 | 27  4.1 | **0.07** | £ ¤ |
| Lentiform nucleus  n ( %) | 182  25.1 | 19  26.0 | 163  25.0 | 0.85 | £ |
| Insular  n ( %) | 39  5.1 | 6  7.7 | 33  4.8 | 0.28 | £ |
| Corona radiata  n ( %) | 220  30.3 | 28  38.4 | 192  29.4 | **0.12** | £ ¤ |
| Corpus callosum  n (%) | 1  0.1 | 1  1.4 | 0 | 0.10 | £ |
| Temporal lobe  n ( %) | 72  9.9 | 10  13.7 | 62  9.5 | 0.26 | £ |
| Frontal lobe  n ( %) | 53  7.3 | 5  6.8 | 48  7.4 | 0.87 | £ |
| Parietal lobe  n ( %) | 35  4.8 | 3  4.1 | 32  4.9 | 1.0 | £ |
| Cccipital lobe  n ( %) | 57  7.9 | 4  5.5 | 53  8.1 | 0.43 | £ |
| Brainstem  n (%) | 121  15.8 | 10  12.8 | 111  16.2 | 0.52 | £ |
| Cerebellum  n ( %) | 48  6.3 | 1  1.3 | 47  6.8 | 0.08 | £ |
| Hemorrhagic transformation  n ( %) | 6  0.8 | 2  2.7 | 4  0.6 | **0.03** | £ ¤ |
| *Corresponding extracranial ICA Characteristics* | | | | | |
| Stenosis 50-99%  n ( %) | 32  4.4 | 4  5.5 | 28  4.3 | 0.55 | £ |
| Occlusion  n ( %) | 11  1.5 | 4  5.5 | 7  1.1 | **0.02** | £ ¤ |
| *Corresponding Intracranial Artery Characteristics* | | | | | |
| Stenosis 50-99%  n ( %) | 57  7.9 | 4  5.5 | 53  8.1 | 0.43 | £ |
| Occlusion  n ( %) | 60  8.3 | 10  13.7 | 50  7.7 | **0.06** | £ ¤ |
| *Corresponding intracranial artery occlusion site* | | | | | |
| ICA  n ( %) | 14  1.9 | 0 | 14  2.1 | 0.38 | £ |
| MCA  n ( %) | 33  4.6 | 9  12.3 | 24  3.7 | **< 0.01** | £ ¤ |
| ACA  n ( %) | 1  0.1 | 0 | 1  0.2 | 1.0 | £ |
| PCA  n ( %) | 6  0.8 | 0 | 6  1.0 | 1.0 | £ |
| BA  n ( %) | 6  0.8 | 1  1.4 | 5  0.8 | 0.47 | £ |

£ - chi-square test

¤ - variable that satisfies p < 0.2 and has clinical value that may be related to END.

ICA: Internal Carotid Artery; ACA: Anterior Cerebral Artery; MCA: Middle Cerebral Artery; PCA; Posterior Cerebral Artery; BA: Basilar Artery;

We discovered that the END group had a higher rate of ischemic stroke site in the internal capsule, hemorrhagic transformation rate, extracranial carotid artery occlusion rate, middle cerebral artery occlusion rate, and intracranial artery occlusion than (**Table 3**).

**Table 4** **The time window when END occurs**

| **The time window when END occurs**  (n=88) |  |
| --- | --- |
| < 4,5h  n ( %) | 15 (17.1 %) |
| 4,5-24h  n ( %) | 23 (26.1 %) |
| 24-48h  n ( %) | 31 (35.2%) |
| 48-72h  n ( %) | 19 (21.6%) |

In the END group, we found that most of the patients had complications within the first 24 hours of the disease, accounting for 43.2% (of which the first 4.5-hour window accounted for 17.1% and the first 4.5-24-hour window accounted for 26.1%), the 24-48-hour window accounted for 35.2%, and the 48-72-hour window accounted for 21.6% (**Table 4**).

**Table 5** **NIHSS score increases when END occurs**

| **NIHSS score increases when END occurs**  (n=88) |  |
| --- | --- |
| 2  n ( %) | 37 (42.1 %) |
| 3  n ( %) | 17 (19.3 %) |
| >=4  n ( %) | 34 (38.6 %) |

At the onset of END, the NIHSS score increased by 2 points in 42.1%; the group with NIHSS increased by 3 points in 19.3% and increased by 4 points or more in 38.6% (**Table 5**).

**Table 6** **Multivariate logistic regression analysis of some prognostic factors for END**

| CLINICAL CHARACTERISTICS | p  OR (95% CI) |
| --- | --- |
| Male | 0.60 ^a^  1.14 (0.70 - 1.86) |
| SBP | 0.357 ^a^  1.00 (0.99 - 1.02) |
| DBP | 0.77 ^a^  1.00 (0.98 - 1.03) |
| SBP150 | **0.04 ^c^**  **1.70 (1.03 - 2.81)** |
| NIHSS | **0.02 ^b^**  **1.24 (1.03 - 1.49)** |
| Hypertension history | 0.33 ^a^  1.34 (0.74 - 2.41) |
| BLOOD TEST CHARACTERISTICS | |
| Platelets | 0.182 ^a^  1.00 (0.99 - 1.01) |
| Fibrinogen | 0.37 ^a^  1.15 (0.85 - 1.55) |
| Cholesterol | 0.57 ^a^  1.10 (0.79 - 1.53) |
| LDL-C | 0.77 ^a^  1.07 (0.71 - 1.61) |
| GPT | 0.24 ^a^  0.99 (0.97 - 1.01) |
| Glucose on admission | **0.02 ^c^**  **1.07 (1.01 - 1.14)** |
| TREATMENT CHARACTERISTICS | |
| Reperfusion treatment | **< 0.01 ^c^**  **3.35 (1.50 - 7.49)** |
| Antiplatelet monotherapy | **< 0.01 ^c^**  **3.69 (2.24 - 6.08)** |
| Antiplatelet dual therapy | 0.63 ^a^  1.67 (0.21 - 13.23) |
| IMAGE CHARACTERISTICS | |
| Internal capsule | **< 0.01 ^d^**  **2.54 (1.37 - 4.71)** |
| Caudate nucleus | 0.12 **^d^**  2.11 (0.83 - 5.31) |
| Corona radiata | 0.13 **^d^**  1.50 (0.89 - 2.53) |
| Hemorrhagic transformation | **0.04 ^f^**  **5.72 (1.07 - 30.45)** |
| Corresponding extracranial ICA Occlusion | **0.02 ^d^**  **4.84 (1.26 - 18.65)** |
| Corresponding Intracranial Artery Occlusion | 0.24 ^e^  1.606 (0.73 - 3.55) |
| MCA Occlusion | **0.01 ^d^**  **3.06 (1.29 - 7.30)** |

1. Adjusted for sex, initial SBP, initial DBP, initial NIHSS, hypertension, platelets, fibrinogen, cholesterol, LDLC, GPT, glucose on admission, reperfusion treatment, antiplatelet monotherapy, antiplatelet dual therapy.
2. Adjusted for sex, initial SBP150, initial NIHSS, hypertension, platelets, fibrinogen, and glucose on admission.
3. Adjusted for sex, initial SBP150, initial NIHSS, hypertension, platelets, fibrinogen, glucose on admission, reperfusion treatment, and antiplatelet monotherapy.
4. Adjusted for ischemic stroke in the internal capsule, ischemic stroke in the caudate nucleus, ischemic stroke in corona radiata, hemorrhagic transformation, MCA occlusion, and corresponding extracranial ICA occlusion.
5. Adjusted for internal capsule, caudate nucleus, corona radiata, hemorrhagic transformation, corresponding extracranial ICA occlusion, and corresponding intracranial artery occlusion.
6. Adjusted for hemorrhagic transformation, glucose on admission, reperfusion treatment, initial SBP150, and initial NIHSS.

SBP – systolic blood pressure; DBP – diastolic blood pressure; NIHSS – national institute of health stroke scale; SBP150 – systolic blood pressure on admission more than 150mmHg; LDLC – low-density lipoprotein; GPT – glutamic pyruvic transaminase; ICA - Internal Carotid Artery; ACA - Anterior Cerebral Artery; MCA - Middle Cerebral Artery

We conducted a multivariate logistic regression analysis to identify some prognostic factors for END in the group of patients with minor stroke, confirming NIHSS score at admission, SBP more than 150mmHg at admission, blood glucose at admission, reperfusion therapy (including thrombolysis and mechanical thrombectomy), use of single antiplatelet drugs, ischemic stroke at the internal capsule location, hemorrhagic transformation (**Table 5**).

**Table 7:** **END & mRS 2-6 at discharge, 30^th^ day and 90^th^ day**

| mRS | p | OR | 95% CI | |
| --- | --- | --- | --- | --- |
|  |  |  | **Lower** | **Upper** |
| At discharge | <0.01 | 22.76 | 11.22 | 46.20 |
| 30^th^ day | <0.01 | 24.38 | 14.40 | 41.29 |
| 90^th^ day | <0.01 | 21.74 | 12.63 | 37.43 |

END was associated with a higher likelihood of poor neurological outcomes (mRS 2-6) at discharge (OR 22.76; 95% CI 11.22-46.20; p<0.01), 30^th^ day of illness (OR 24.38; 95% CI 14.40-41.29; p<0.01), and 90^th^ day of disease (OR 21.74; 95% CI 12.63-37.43; p<0.01).

**OR (95% CI)**

21.74 (12.63-37.43)

24.38 (14.40-41.29)

22.76 (11.22-46.20)

**Figure 1** **END & mRS 2-6 at discharge, 30^th^ day and 90^th^ day**

**Table 8.** **DEFINITION OF VARIABLES**

| **Numer order** | **Variable** | **Define** |
| --- | --- | --- |
|  | Hypertension | Patients are either receiving treatment or have a history of hypertension as defined by WHO 2003 standards (systolic blood pressure ≥ 140 mmHg and/or diastolic blood pressure ≥ 90 mmHg). |
|  | Diabetes type 1&2 | Patients receive diabetes treatment or fulfill the 1999 WHO diagnostic criteria, including a fasting glucose level of ≥ 7 mmol/l. |
|  | Cigarette Smoking | Patients who smoked one cigarette daily for one year before experiencing a stroke. |
|  | Cardiovascular disease | Coronary artery disease, heart failure (EF < 55%), myocardial infarction or peripheral arterial disease |
|  | History of ischemic stroke/TIA | Individuals with a history of ischemic stroke or transient ischemic attack (TIA) |
|  | Atrial Fibrillation | Patients admitted with a diagnosis of atrial fibrillation or with a history of chronic or paroxysmal atrial fibrillation. |
|  | Dyslipidemia | Patients undergoing treatment or total care have Cholesterol levels of 5 mmol/l or higher and LDL levels of 3 mmol/l or higher. |
|  | Overweight/Obesity | Individuals with a Body Mass Index (BMI) of 25 or greater, as defined by the World Health Organization (WHO). |
|  | Mechanical heart valve | Individuals with a history of mechanical heart valve replacement surgery |
|  | Bioprosthetic heart valve | Individuals with a history of biological heart valve replacement surgery |
|  | Moderate to severe mitral stenosis | Individuals with a history of moderate to severe mitral stenosis |
|  | mRS background | Patients with prior modified Rankin scale disability scores |

***Conference***: Putaala J, Metso AJ, Metso TM, et al. Analysis of 1008 consecutive patients aged 15 to 49 with first-ever ischemic stroke: the Helsinki young stroke registry. Stroke. 2009 Apr;40(4):1195-1203.
